# Changes of Neural Pathways after Vojta Approach in a Child with Developmental Delay

**DOI:** 10.3390/children8100918

**Published:** 2021-10-15

**Authors:** Sun-Young Ha, Yun-Hee Sung

**Affiliations:** 1 Department of Physical Therapy, Graduate School, Kyungnam University, Changwon 51767, Korea; mallows205@naver.com; 2 Department of Physical Therapy, College of Health Sciences, Kyungnam University, Changwon 51767, Korea

**Keywords:** developmental delay, Vojta approach, corticospinal tract, corticoreticular pathway, gross motor

## Abstract

The development of motor function is related to the development of neural pathways in the white matter. Children with developmental delay (DD) and hypotonia have reduced motor function, and their neural pathways are observed differently from those of typically developed children. We investigated changes in neural pathways through diffusion tensor imaging (DTI) after utilizing the Vojta approach. The participant was a child with DD and hypotonia, and had delayed motor function. Although he had no brain damage on magnetic resonance imaging findings, damage to the neural pathway was confirmed through DTI due to cytomegalovirus infection in the mother’s womb. From 11 months of age, the Vojta approach was performed for a total of 8 months. In this study, we found that in CST, the left FA and right TV increased in follow-up DTI more than in the initial DTI. In CRP, Wallerian degeneration was observed in the left FA, MD, and TV in follow-up DTI. GMFM-88 improved after intervention. The structural change of neural pathways through the Vojta approach influenced the improvement of gross motor function. Therefore, it is thought that the Vojta approach can be suggested as a meaningful intervention for children with DD and hypotonia.

## 1. Introduction

The Vojta approach induces a certain movement by stimulating specific stimulation zones. It is a method that suppresses abnormal movement, and induces normal motor development by repeatedly memorizing the normal movement in the brain [1]. These stimuli suppress the patient’s wrong compensatory movements, and promote correct postural control [2]. Therefore, the Vojta approach is used as an intervention method for spastic cerebral palsy (CP) [3], developmental delay (DD) [4], and musculoskeletal diseases [5].

DD and hypotonia are diseases that cause problems due to interference with normal brain development at the time of brain formation, or after birth [6]. This includes various diseases such as chromosomal diseases, high-risk infants, and various syndromes. They are characterized by delayed motor development [7].

The development of motor function is related to the development of neural pathways [8]. The neural pathways involved in motor function include the corticospinal tract (CST) and the non-corticospinal tract (non-CST), located in the white matter [9]. The CST tract plays a crucial role in fine motor functions, such as voluntary movement control of the extremities and delicate hand movements [8,10]. Among the non-CST, the corticoreticular pathway (CRP) is mainly involved in the trunk and axial muscles, and affects gross motor function such as trunk control and gait [8,11]. Children with DD and hypotonia have a smaller white matter volume than typically developed children, showing abnormal myelination [12]. Moreover, the neural pathways located in the white matter are observed differently from typically developed children [13,14]; these problems are related to the motor development of children.

Diffusion tensor imaging (DTI) is a method for evaluating the structure of these neural pathways, which is an effective test for studying the fundamental structure of white matter [15]. DTI is a non-invasive test that detects and analyzes changes in movement in a certain direction, using the property of water molecules to diffuse in white matter [15]. The findings of lesions in children with DD and hypotonia vary, and they may cause DD despite normal magnetic resonance imaging (MRI) findings. Clinically applied rehabilitation improves motor function and brain microstructure [16], but it is not easy to demonstrate the mechanism by which the improvement occurs. DTI can evaluate the lesions of the neural pathways of the white matter [17], and demonstrate deranged myelination [12]. In addition, it is possible to estimate the improvement of motor function through changes to the CST and CRP [18].

Among the various intervention methods, the Vojta approach is reported to be effective in improving the motor function of children with DD and hypotonia [4], but studies on structural changes in the neural pathways have not been reported. Therefore, the purpose of this study was to investigate the effect of the Vojta approach on the CST and CRP changes in children with DD and hypotonia.

## 2. Materials and Methods

### 2.1. Case Presentation

The participant was a male, born on 19 April 2016, with a gestation period of 39 weeks and 4 days, and a birth weight of 2.6 kg. At birth, his heart rate decreased, and autonomous respiration was difficult, so oxygen therapy was given for 10 days. MRI showed no brain damage. Thereafter, he was discharged from the hospital and lived at home, and there were no special features in sleep, diet, and other lifestyle patterns. However, he had difficulty in head control and eye contact until approximately six months of age, and was diagnosed with DD at the hospital in October 2016.

In February 2017, there was no progress in motor development, so he underwent a thorough examination at the hospital. As a result, the child was diagnosed with cytomegalovirus infection transmitted from the mother in utero. He was hospitalized for approximately one month for cytomegalovirus treatment, and was discharged after completion of treatment. While in the hospital, he underwent an initial DTI at 10 months of age, which confirmed damage to his neural pathways. He also had hearing problems. He was discharged in early March 2017, and was able to control his head and roll over at the time. The child received the Vojta approach from early March 2017, after his hospital discharge. A follow-up DTI was performed after 9 months. The goal of rehabilitation for the child and parent involved was to enable normal motor development and participation in daily life through early intervention. We informed the child and parent of the purpose of the study, and they consented to this study. This study was approved by the Research Ethics Committee of Kyungnam University (1040460-A-2020-055).

### 2.2. Diffusion Tensor Imaging (DTI)

DTI was used to measure the neural pathway changes before (10 months of age) and after (18 months of age) intervention application. Since the child was young and unable to follow the examiner’s verbal instructions, the child was tested after taking a sleep-inducing drug to limit movement during the measurement.

The DTI data were acquired using a Synergy-L Sensitivity Encoding (SENSE) head coil on a 1.5 T Philips Gyroscan Intera (Philips, Best, The Netherlands), and single-shot echo-planar imaging (EPI). For each of the 32 non-collinear and non-coplanar diffusion-sensitizing gradients, we acquired 60 contiguous slices parallel to the anterior commissure–posterior commissure line. The image parameters were as follows: matrix = 128 × 128; field of view = 221 × 221 mm^2^; TE = 76 ms; TR = 10.726 ms; SENSE factor = 2, EPI factor = 67; b = 600 mm^2^ s^−1^; NEX = 1; and a slice thickness of 2.3 mm. Pre-processing of the DTI data set was performed using the Oxford Centre for Functional Magnetic Resonance Imaging of the Brain (FMRIB) software library (FSL), and DTI Studio software (CMRM, Johns Hopkins Medical Institute, Baltimore, MD, USA) was used for reconstruction of CST and CRP.

The fiber tracking was performed using a fractional anisotropy (FA) threshold of >0.15 and direction threshold of <70°. The three-dimensional fibers were superimposed on the T2 transverse images performed immediately after DTI imaging. We measured the FA, mean diffusion (MD), and tract volume (TV) of CST and CRP in both hemispheres [19].

### 2.3. Gross Motor Function

The Gross Motor Function Measure-88 (GMFM-88) is a standardized evaluation tool that can evaluate changes in gross motor function and the effects of treatment [20]. The items are divided into 5 areas: A (lying and rolling), B (sitting), C (crawling and kneeling), D (standing), and E (walking, running, and jumping). The validity of GMFM-88 is 0.91 [21], the inter-rater reliability is 0.77, the test-retest reliability is 0.88, and the intra-rater reliability is 0.68 [22]. GMFM-88 was measured before and after intervention.

### 2.4. Intervention

Intervention was performed by physical therapist. The Vojta approach consists of reflex turning 1, reflex turning 2, and reflex creeping. Reflex turning 1 and reflex turning 2 target the quadrupedal position by stimulating specific zones, and the reflex creeping induces forward movement from the prone position. The intervention was applied for 40 min per session, 3 times a week, for a total of 8 months. It consisted of 10 min each, depending on posture, a total of 30 min, and stretching was performed for 5 minutes before and after the intervention.

## 3. Results

### 3.1. Change of the Neural Pathway

In the CST, the right FA decreased at follow-up rather than at the initial intervention, but the left FA increased at follow-up rather than at the initial intervention. The right and left MD decreased at follow-up rather than at the initial intervention. The right TV increased at follow-up rather than at the initial intervention, but the left TV decreased at follow-up rather than at the initial intervention.

**Figure 1:**
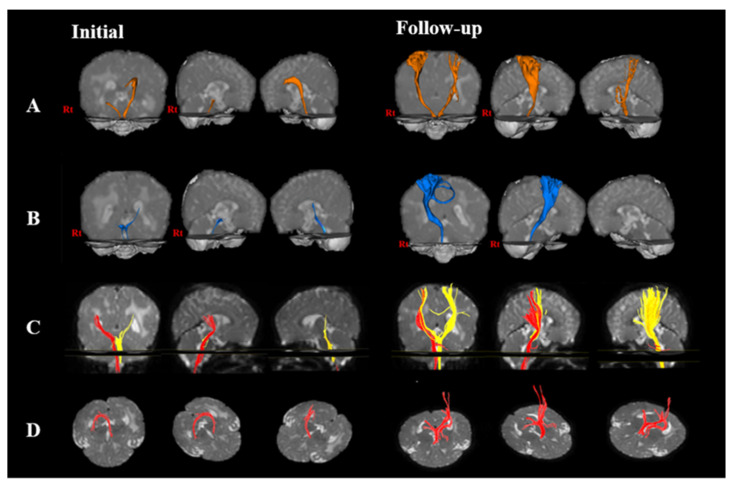
Changes in neural pathway through diffusion tensor imaging (DTI) of a child at initial and follow-up interventions. The initial DTI was performed at 10 months of age, and the follow-up DTI was performed after the intervention was applied for 8 months. (**A**) corticospinal tract; (**B**) corticoreticular pathway; (**C**) medial lemniscus; and (**D**) fornix.

In the CRP, the right FA decreased at follow-up than at the initial intervention, but the right MD and TV increased at follow-up rather than at the initial intervention. The left FA, MD, and TV showed degeneration at the initial intervention (Figure 1) (Table 1). 

### 3.2. Change of the Gross Motor Function

In the GMFM-88 scale, gross motor function improved from 17.17% at initial intervention to 57.35% at follow-up (Table 2).

## 4. Discussion

Myelination of white matter is closely related to the development of sensory, motor, and cognitive functions in children [23,24], and children with DD are observed to decrease FA and increase MD during DTI [13]. The purpose of this study was to investigate the effect of the Vojta approach on the neural pathway changes of white matter in children with DD and hypotonia.

The development of the CST affects the prognosis of motor function [23]. Azizi et al. showed that when anti-gravity treadmill training was applied to children with CP, FA increased and MD decreased in the CST, and gait function was improved [18]. They reported that the intervention could induce neuroplasticity by positively affecting the structure and motor function of neural pathways. Moreover, Trivedi et al. showed that, when DTI were compared after 6 months of rehabilitation in spastic quadriplegia [25], FA of the CST was increased, indicating that the improved clinical status demonstrates plasticity in the cortex. In this study, the increase of FA in the CST and improvement of motor function after applying the Vojta approach demonstrates increased plasticity in the CST. An increase in TV in the right side also contributes to the maturation of more precise fine motor skills, such as motor timing, and motor integration [8,26,27]. In this study, the increase of TV in the right CST is thought to affect the precise movement of the left hand. Therefore, stimulation of the specific zones through the Vojta approach is thought to have affected the neuroplasticity by communicating the inhibited movement to the central nervous system.

The CRP exhibits symmetrical integrity during normal motor development, but if disruption occurs, it cannot be considered normal development [8]. As a result of this study, FA of the right CRP was decreased, MD and TV were increased, and the left side showed degeneration. The therapeutic effect for improving motor function was more correlated with TV than FA in DTI analysis [28]. Rha et al. reported that the high-functioning CP group showed higher TV than the low-functioning CP group, but that there was no difference in FA between the two groups [29]. In the study of Kwon et al., the bilateral trunk instability group among children with DD showed lower FA and TV in the bilateral CRP. On the other hand, the unilateral trunk instability group showed lower FA and TV in the CRP, opposed to the clinical signs of typically developed children [30]. Moreover, in a study by Son and Shin, due to observing DTI in children with trunk weakness, although MRI did not show any clear abnormal lesions, the CRP showed a difference between the severely damaged side and the less damaged side [31]. In studies by Kwon et al. and Son and Shin, it was reported that the disruption to the CRP, even though there was no lesion in the MRI results in common, was due to microstructural damage or pathological immaturity [30,31]. In this study, TV of the right CRP increased after the intervention, which is thought to influence the improvement of trunk function and gait. However, in the left CRP, Wallerian degeneration was observed. These results suggest that microstructural damage caused by cytomegalovirus infection in the child affected the neuronal degeneration. It is thought that microstructural damage also affected the right side, resulting in a decrease in FA, and an increase in MD on the right side. Nevertheless, the child was able to walk independently by 2–3 steps around the time of DTI imaging at follow-up, which is thought to be because the increase in TV of the CRP affected the control of the contralateral proximal and bilateral axial muscles [32]. In addition, the increase in TV of the right CRP is thought to be an effect of the Vojta approach applied early, and the study of Jang et al. which supports our results demonstrates that the increase in TV of the CRP affects the improvement of gait and motor function [33].

The limitation of this study is that it is a single case report, and it is difficult to generalize the results, since there is only one participant. Furthermore, there was no control group, so it was difficult to compare the results. In addition, it was difficult to perform various functional evaluations due to the young age of the subject. Therefore, in future work, studies that complement these limitations should be conducted.

## 5. Conclusions

We were able to objectively prove the effect of the Vojta approach on the improvement of gross motor function by observing neural pathway changes using DTI in a child with DD and hypotonia.

## Figures and Tables

**Table 1:** Changes in CST and CRP pre- and post-intervention.

		CST		CRP	
		Initial	Follow-Up	Initial	Follow-Up
FA	Right	0.263	0.330	0.350	0.290
	Left	0.330	0.398	0.307	-
MD	Right	1.520	1.006	0.943	0.002
	Left	1.110	0.987	0.012	-
TV	Right	244.000	2508.000	152.000	183.000
	Left	507.000	354.000	3920.000	-

CST: corticospinal tract; CRP: corticoreticular pathway; FA: fractional anisotropy; MD: mean diffusion; TV: track volume.

**Table 2:** Changes in gross motor function (unit: %).

	Initial	Follow-Up
GMFM-88	17.17	57.35

GMFM-88: gross motor function measure-88.

## Data Availability

All relevant data are within the manuscript.

## References

[1] Khan M.H., Helsper J., Farid M.S., Grzegorzek M. (2018). A computer vision-based system for monitoring Vojta therapy. Int. J. Med. Inform..

[2] Epple C., Maurer-Burkhard B., Lichti M.C., Steiner T. (2020). Vojta therapy improves postural control in very early stroke rehabilitation: A randomised controlled pilot trial. Neurol. Res. Pract..

[3] Sung Y.-H., Ha S.-Y. (2020). The Vojta approach changes thicknesses of abdominal muscles and gait in children with spastic cerebral palsy: A randomized controlled trial, pilot study. Technol. Health Care.

[4] Giannantonio C., Papacci P., Ciarniello R., Tesfagabir M.G., Purcaro V., Cota F., Semeraro C.M., Romagnoli C. (2010). Chest physiotherapy in preterm infants with lung diseases. Ital. J. Pediatr..

[5] Jung M.W., Landenberger M., Jung T., Lindenthal T., Philippi H. (2017). Vojta therapy and neurodevelopmental treatment in children with infantile postural asymmetry: A randomised controlled trial. J. Phys. Ther. Sci..

[6] Widjaja E., Nilsson D., Blaser S., Raybaud C. (2008). White matter abnormalities in children with idiopathic developmental delay. Acta Radiol..

[7] Hong B.Y., Jo L., Kim J.S., Lim S.H., Bae J.M. (2017). Factors Influencing the Gross Motor Outcome of Intensive Therapy in Children with Cerebral Palsy and Developmental Delay. J. Korean Med. Sci..

[8] Yeo S.S., Jang S.H., Son S.M. (2014). The different maturation of the corticospinal tract and corticoreticular pathway in normal brain development: Diffusion tensor imaging study. Front. Hum. Neurosci..

[9] De Oliveira-Souza R. (2012). The human extrapyramidal system. Med. Hypotheses.

[10] Son S.M., Park S.H., Moon H.K., Lee E., Ahn S.H., Cho Y.W., Byun W.M., Jang S.H. (2009). Diffusion tensor tractography can predict hemiparesis in infants with high risk factors. Neurosci. Lett..

[11] Matsuyama K., Mori F., Nakajima K., Drew T., Aoki M., Mori S. (2004). Locomotor role of the corticoreticular-reticulospinal-spinal interneuronal system. Prog. Brain Res..

[12] Pujol J., López-Sala A., Sebastián-Gallés N., Deus J., Cardoner N., Soriano-Mas C., Moreno A., Sans A. (2004). Delayed myelination in children with developmental delay detected by volumetric MRI. Neuroimage.

[13] Filippi C.G., Lin D.D., Tsiouris A.J., Watts R., Packard A.M., Heier L.A., Uluğ A.M. (2003). Diffusion-tensor MR imaging in children with developmental delay: Preliminary findings. Radiology.

[14] Verma A., Sagar N.C., Kumar A., Srivastava A. (2015). Diagnostic value of diffusion tensor imaging derived metrics as biomarkers of cerebral changes in developmental delay. Indian J. Radiol. Imaging.

[15] Ment L.R., Hirtz D., Hüppi P.S. (2009). Imaging biomarkers of outcome in the developing preterm brain. Lancet Neurol..

[16] Bleyenheuft Y., Dricot L., Gilis N., Kuo H.C., Grandin C., Bleyenheuft C., Gordon A.M., Friel K.M. (2015). Capturing neuroplastic changes after bimanual intensive rehabilitation in children with unilateral spastic cerebral palsy: A combined DTI, TMS and fMRI pilot study. Res. Dev. Disabil..

[17] Chang M.C., Jang S.H., Yoe S.S., Lee E., Kim S., Lee D.G., Son S.M. (2012). Diffusion tensor imaging demonstrated radiologic differences between diplegic and quadriplegic cerebral palsy. Neurosci. Lett..

[18] Azizi S., Birgani P.M., Marzbani H., Nourian R., Kohanpour M., Mirbagheri M.M. (2018). Assessment of neuroplasticity of corticospinal tract induced by antigravity treadmill (AlterG) in cerebral palsy children. Annu. Int. Conf. IEEE Eng. Med. Biol. Soc..

[19] Jones D.K., Knösche T.R., Turner R. (2013). White matter integrity, fiber count, and other fallacies: The do’s and don’ts of diffusion MRI. Neuroimage.

[20] Park E.S., Rha D.W., Shin J.S., Kim S., Jung S. (2014). Effects of hippotherapy on gross motor function and functional performance of children with cerebral palsy. Yonsei Med. J..

[21] Palisano R.J., Hanna S.E., Rosenbaum P.L., Russell D.J., Walter S.D., Wood E.P., Raina P.S., Galuppi B.E. (2000). Validation of a model of gross motor function for children with cerebral palsy. Phys. Ther..

[22] Nordmark E., Hägglund G., Jarnlo G.B. (1997). Reliability of the gross motor function measure in cerebral palsy. Scand. J. Rehabil. Med..

[23] Drobyshevsky A., Bregman J., Storey P., Meyer J., Prasad P.V., Derrick M., MacKendrick W., Tan S. (2007). Serial diffusion tensor imaging detects white matter changes that correlate with motor outcome in premature infants. Dev. Neurosci..

[24] Nagy Z., Westerberg H., Klingberg T. (2004). Maturation of white matter is associated with the development of cognitive functions during childhood. J. Cogn. Neurosci..

[25] Trivedi R., Gupta R.K., Shah V., Tripathi M., Rathore R.K., Kumar M., Pandey C.M., Narayana P.A. (2008). Treatment-induced plasticity in cerebral palsy: A diffusion tensor imaging study. Pediatr. Neurol..

[26] Froehle A.W., Nahhas R.W., Sherwood R.J., Duren D.L. (2013). Age-related changes in spatiotemporal characteristics of gait accompany ongoing lower limb linear growth in late childhood and early adolescence. Gait Posture.

[27] Mayson T.A., Harris S.R., Bachman C.L. (2007). Gross motor development of Asian and European children on four motor assessments: A literature review. Pediatr. Phys. Ther..

[28] Kim J.H., Kwon Y.M., Son S.M. (2015). Motor function outcomes of pediatric patients with hemiplegic cerebral palsy after rehabilitation treatment: A diffusion tensor imaging study. Neural Regen. Res..

[29] Rha D.W., Chang W.H., Kim J., Sim E.G., Park E.S. (2012). Comparing quantitative tractography metrics of motor and sensory pathways in children with periventricular leukomalacia and different levels of gross motor function. Neuroradiology.

[30] Kwon Y.M., Rose J., Kim A.R., Son S.M. (2017). Corticoreticular tract lesion in children with developmental delay presenting with gait dysfunction and trunk instability. Neural Regen. Res..

[31] Son S.M., Shin S.M. (2017). Disruption of the Corticoreticular Tract in Pediatric Patients with Trunk Instability: A Diffusion Tensor Tractography Study. Ann. Rehabil. Med..

[32] Halsband U., Ito N., Tanji J., Freund H.J. (1993). The role of premotor cortex and the supplementary motor area in the temporal control of movement in man. Brain.

[33] Jang S.H., Chang C.H., Lee J., Kim C.S., Seo J.P., Yeo S.S. (2013). Functional role of the corticoreticular pathway in chronic stroke patients. Stroke.

